# Impact of Gastrointestinal Symptoms on Quality of Life in Individuals With Chronic Kidney Disease in Cameroon: A Hospital-Based Cross-Sectional Study

**DOI:** 10.7759/cureus.82427

**Published:** 2025-04-17

**Authors:** Rostand Dimitri Messanga Bessala, Vuchichi Boris Vugugah, Erna Hosanna Ngo Kam

**Affiliations:** 1 Public Health, Newcastle University, Newcastle Upon Tyne, GBR; 2 Internal Medicine, Faculty of Medicine and Biomedical Sciences, University of Yaounde I, Yaounde, CMR

**Keywords:** adults, cameroon, chronic kidney disease, gastrointestinal quality of life index, gastrointestinal symptoms

## Abstract

Introduction

Gastrointestinal symptoms (GIS) are commonly reported in individuals with chronic kidney disease (CKD) and can significantly impact quality of life (QoL). Despite this, data from sub-Saharan Africa remain scarce. This study aimed to evaluate the effect of GIS on QoL in CKD patients in Cameroon.

Materials and methods

A cross-sectional study was carried out between December 2021 and May 2022 at the Yaounde General Hospital and Yaounde University Teaching Hospital. Participants included adults (≥18 years) with CKD stages III-V or on hemodialysis. GIS were assessed using a Modified Rome IV Diagnostic Questionnaire, and QoL was measured with the Gastrointestinal Quality of Life Index (GIQLI). Associations and correlations between GIS, QoL, and clinical characteristics were analyzed using Spearman's correlation and multiple regression, with significance set at p<0.05.

Results

Among 202 participants (median age 58 years, 63.4% male), 82.2% reported at least one GIS. The most common symptoms were anorexia (41.1%), constipation (39.6%), nausea (36.6%), bloating (33.7%), and vomiting (32.2%). QoL showed a weak negative correlation with serum creatinine (rho=-0.36; p<0.001) and a weak positive correlation with estimated glomerular filtration rate (eGFR) (rho=0.40; p<0.001). GIS and hypertension were significantly associated with lower QoL scores. Specifically, the presence of GIS (aOR=0.17; CI: 0.06-0.40; p<0.001) and hypertension (aOR=0.35; CI: 0.13-0.85; p=0.02) reduced the odds of better QoL.

Conclusion

GIS are prevalent among CKD patients in Cameroon and are linked to diminished QoL. Reduced renal function and hypertension further compound this effect. Routine QoL assessments and symptom-targeted interventions should be integrated into clinical care to enhance patient outcomes.

## Introduction

Chronic kidney disease (CKD) is a major global health issue, affecting approximately one in 10 individuals worldwide [1,2]. It is characterized by a progressive decline in renal function, indicated by a glomerular filtration rate (GFR) of less than 60 ml/min/1.73 m², the presence of markers of renal damage, or both, persisting for at least three months [3,4]. The disease typically progresses slowly and often remains asymptomatic until it reaches advanced stages. CKD has widespread effects on multiple organ systems, including the gastrointestinal tract [4]. Gastrointestinal symptoms (GIS) such as anorexia, nausea, and constipation are common in CKD patients, but are frequently underreported [5,6]. The true prevalence of these symptoms remains uncertain, with reported rates varying widely, ranging from as low as 32% to as high as 80% [7]. The pathophysiology of GIS in CKD is complex and not fully understood. Several factors, including the baseline kidney disease, uremia, high prevalence of anxiety disorders, renal replacement therapy, alterations in the microbiota, and increased serum levels of various gastrointestinal hormones, are known to contribute to these symptoms [5]. These symptoms tend to worsen as renal function declines and may or may not improve with the initiation of renal replacement therapy. Regardless of their progression, GIS are known to negatively impact the quality of life (QoL) of CKD patients [8-10].

According to the World Health Organization (WHO), QoL is defined as "an individual's perception of their position in life in the context of the culture and value systems in which they live and in relation to their goals, expectations, standards, and concerns" [11]. While numerous studies have examined health-related QoL across the spectrum of CKD, most have used generic tools such as the Survey Form-36 (SF-36) and EuroQol Five Dimension (EQ-5D). Few studies, however, have explored the influence of GIS on QoL. To our knowledge, limited research has focused on GIS-related QoL using the Gastrointestinal Quality of Life Index (GIQLI). Moreover, research on this topic is scarce in sub-Saharan Africa. Understanding how GIS affect QoL in this region is crucial for improving clinical management and patient outcomes. Incorporating gastrointestinal QoL assessments into routine care for individuals with CKD could enhance symptom recognition and guide more targeted interventions. Therefore, this study was conducted with the primary objective of assessing the impact of GIS on QoL in CKD patients in Cameroon. Specifically, it sought to determine the frequency of GIS, evaluate their impact on QoL, and identify determinants of QoL in CKD patients.

## Materials and methods

Study design

A hospital-based cross-sectional study was conducted from December 2021 to May 2022.

Setting

The study was carried out at two hospitals: Yaounde General Hospital (YGH) and Yaounde University Teaching Hospital (YUTH). These facilities serve as major nephrology centers in the region, providing care to patients with CKD. CKD patients on hemodialysis receive two weekly in-center sessions of four hours each.

Inclusion criteria

Participants aged 18 years or older with a confirmed diagnosis of CKD stages III-V or those undergoing hemodialysis were included in the study. CKD stages were defined according to the Kidney Disease Improving Global Outcomes (KDIGO) 2012 guidelines [12].

Exclusion criteria

Participants with cognitive impairments, with acute illness, or who had undergone a recent abdominal procedure were excluded from the study.

Variables

Demographic data and comorbidities for each participant were obtained from medical records. Demographic information included age at study entry, sex, baseline nephropathy, duration on hemodialysis, body mass index (BMI), serum creatinine levels, and renal function, which was assessed using the estimated glomerular filtration rate (eGFR). The eGFR for CKD stages III-V was estimated using the Modification of Diet in Renal Disease (MDRD) formula. Comorbidities, including diabetes and hypertension, were also recorded. GIS were assessed using a modified version of the Rome IV Diagnostic Questionnaire (R4DQ) (Appendix 1). A subset of symptoms, including anorexia, constipation, diarrhea, nausea, vomiting, bloating, epigastric pain, inappetence, and dysgeusia, was selected based on their clinical relevance to CKD and study feasibility. The R4DQ is a self-administered questionnaire developed specifically to assess functional gastrointestinal disorders [13]. QoL was evaluated using the GIQLI, a validated tool designed to assess QoL in patients with gastrointestinal diseases or symptoms (Appendix 2). The GIQLI includes 36 items grouped into five domains: core symptoms, physical, psychological, social, and disease-specific [14]. The maximum and minimum total scores are 144 and 0, respectively. Subscores for each question range from 0 to 4, and a GIQLI score of >90 represents a better QoL [14]. Definitions of GIS considered in the study are as follows: Anorexia was defined as a loss of appetite; nausea was defined as a feeling of sickness with the urge to vomit; constipation was defined as hard, difficult-to-pass stools, less than three times a week; bloating was defined as a feeling of abdominal fullness; vomiting was defined as the brutal ejection of all or part of gastric content; diarrhea was defined as the emission of loose or watery stools with a frequency of three or more per day; dysgeusia was defined as an altered taste sensation; inappetence was defined as a sensation of early satiety accompanied by a loss of appetite; and epigastric pain was defined as a discomfort or aching of the epigastric region (upper abdomen). These symptoms were limited to a two-week period prior to enrollment.

Recruitment procedure

Eligible participants were recruited during outpatient consultations or on the day of their hemodialysis sessions. Participant consent forms detailing the study's objectives, potential risks, and participants' rights were provided to all eligible individuals. Informed consent was obtained in writing, and participation in the study was voluntary. Participants could withdraw from the study at any time without affecting their medical care. The questionnaires were researcher-administered, and the primary investigator received a two-day training, including an introduction to the study and its purpose as well as a practical workshop with peers on how to administer the questionnaire. Data were collected using the R4DQ, the GIQLI, and a structured questionnaire that included socio-demographic characteristics, comorbidities, and baseline nephropathy. Pre-testing of the questionnaires was conducted on 10% of the study participants prior to the study's start to ensure the accuracy of data collection. Any errors identified during pre-testing were corrected before the main study was implemented.

Bias

This cross-sectional study may be susceptible to selection bias. To minimize this, participants were recruited from two hospitals, and within each hospital, participants were enrolled from both outpatient consultations and hemodialysis units. Additionally, the questionnaires were pre-tested to ensure consistency in the GIQLI evaluation and to minimize information bias. Furthermore, most questions within the questionnaire focused on the past two weeks, which helped reduce the risk of recall bias. Multivariate analysis was conducted to control for confounding factors in the study population. A trained investigator administered the questionnaires, which helped mitigate interviewer bias.

Study size

Consenting participants who met the eligibility criteria were enrolled. The minimum required sample size was calculated using the single proportion formula [15] n=Z^2^pq/e^2^, where n is the desired sample size, Z is the critical value for a standard normal distribution set at 1.96 for the 95% confidence interval, P is the estimated proportion of CKD which, according to Aseneh et al. [16], corresponds to 0.14 (14%), q is 1-p, and e is the desired margin of error, fixed at 5% (0.05) for a 95% confidence interval. Hence, n=((1.96)^2^(0.14)(1-0.14))/(0.05)^2 ^which is equal to 185.01 or 185.

Statistical methods

Data were cleaned and input into EpiData Version 3.1 (EpiData Association, Odense, Denmark, Europe). Statistical analysis was performed using R Version 4.1.3 (R Foundation for Statistical Computing, Vienna, Austria). Categorical variables were reported as frequencies and percentages, while continuous variables were expressed as means and standard deviations (SD) for normally distributed data or medians and interquartile range (IQR) for skewed distributions. Chi-squared tests or Fisher's exact tests were used to compare categorical variables. Spearman's correlation coefficient was applied to assess the relationship between non-normally distributed continuous variables. Multiple regression was conducted to identify factors independently associated with gastrointestinal QoL in CKD. A p-value of <0.05 was considered statistically significant.

Ethical concerns

Ethical approval was obtained from the Institutional Ethical Review Board of the Faculty of Medicine and Biomedical Sciences of the University of Yaounde I (approval number: 09/UY1/FMSB/VDRC/CSD). Administrative authorizations to conduct the in-hospital study were obtained from YGH and YUTH. Informed consent was obtained from each participant.

## Results

Participants

**Figure 1:**
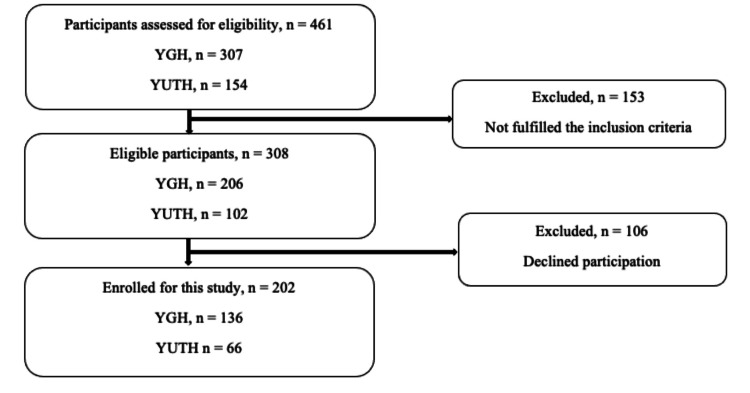
Flowchart of participants' selection YGH: Yaounde General Hospital; YUTH: Yaounde University Teaching Hospital

A total of 202 participants were included in the study, comprising 78 individuals with CKD stages III-V and 124 individuals on hemodialysis. A flowchart illustrating the participant enrollment and exclusion process is provided in Figure 1. The median age of participants was 58 years (IQR: 45.3-66), with 63.4% of participants being male (Table 1). The median serum creatinine level, BMI, and duration on hemodialysis (in months) were 100.2 (IQR: 35-145), 24.8 (IQR: 21.95-28.15), and 24 (IQR: 12-60), respectively. Hypertension was present in 85.1% of the study population (Table 1).

**Table 1:** Participants' demographic characteristics and comorbidities

Variables	Sample (n=202)	Percentage (%)
Age (years)		
15-59	120	59.4
60-85	82	40.6
Sex		
Male	128	63.4
Female	74	36.6
Comorbidity		
Hypertension	172	85.1
Diabetes	68	33.7
Baseline nephropathy		
Hypertensive nephropathy	87	43.1
Diabetic nephropathy	33	16.3

GIS data based on the R4DQ

**Figure 2:**
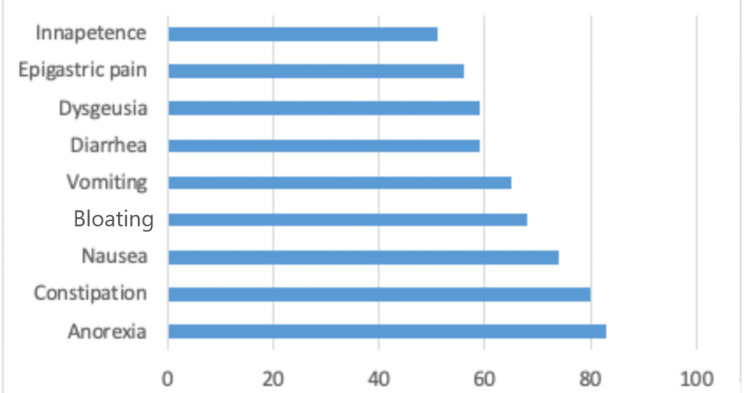
Proportion of gastrointestinal symptoms

Among the 202 participants included in the study, 166 (82.2%) reported experiencing at least one GIS. The most common symptoms were anorexia (41.1%), constipation (39.6%), nausea (36.6%), bloating (33.7%), and vomiting (32.2%) (Figure 2).

A statistically significant association was found between anorexia, nausea, vomiting, diarrhea, dysgeusia, inappetence, and the stage of CKD (p<0.05) (Table 2).

**Table 2:** Gastrointestinal symptoms according to the stage of CKD CKD: chronic kidney disease; *: p-values that are significant, b: p-value of Fisher's exact test Percentages represent the proportion of patients with each symptom across CKD stages. Total (n) is the total number of patients who experienced the symptom.

Symptoms (n)	Frequency (%)					P-value^b^
	CKD3a	CKD3b	CKD4	CKD5ND	CKD5D	
Anorexia (83)	6 (7.2)	1 (1.2)	8 (9.6)	10 (12)	58 (69.9)	0.008^*^
Constipation (80)	5 (6.2)	4 (5)	9 (11.2)	9 (11.2)	53 (66.2)	0.32
Nausea (74)	3 (4.1)	1 (1.4)	8 (10.8)	10 (13.5)	52 (70.3)	0.004^*^
Bloating (68)	4 (5.9)	3 (4.4)	5 (7.4)	9 (13.2)	47 (69.1)	0.055
Vomiting (65)	4 (6.2)	-	4 (6.2)	9 (13.8)	48 (73.8)	0.001^*^
Diarrhea (59)	8 (13.6)	-	4 (6.8)	6 (10.2)	41 (69.5)	0.014^*^
Dysgeusia (59)	3 (5.1)	-	6 (10.2)	10 (16.9)	40 (67.8)	0.003^*^
Epigastric pain (56)	4 (7.1)	2 (3.6)	5 (8.9)	9 (16.1)	36 (64.3)	0.067
Inappetence (51)	3 (5.9)	-	1 (2)	10 (19.6)	37 (72.5)	<0.001^*^

Multivariate analysis (multiple regression) revealed that GIS and hypertension were associated with a lower likelihood of better QoL in patients with CKD (p<0.001; aOR=0.17; CI: 0.06-0.40 for GIS; p=0.02; aOR=0.35; CI: 0.13-0.85 for hypertension) (Table 3).

**Table 3:** Factors associated with quality of life *: statistically significant p-value GIQLI: Gastrointestinal Quality of Life Index; GIS: gastrointestinal symptoms

Variables	GIQLI		P-value	Adjusted OR (CI)
	< median GIQLI	≥ median GIQLI		
Age (years)				
15-59	66 (55)	54 (45)	0.89	1.05 (0.53-2.08)
60-85	38 (46.9)	43 (53.1)		
Sex				
Male	64(50.4)	63(49.6)	0.51	0.81 (0.43-1.51)
Female	40 (54.1)	34 (45.9)		
Hypertension	82 (48)	89 (52)	0.02*	0.35 (0.13-0.85)
Diabetes	33 (48.5)	35 (51.5)	0.68	1.17 (0.56-2.45)
GIS	97 (58.8)	68 (41.2)	<0.001*	0.17 (0.06-0.40)

GIQLI outcomes

**Figure 3:**
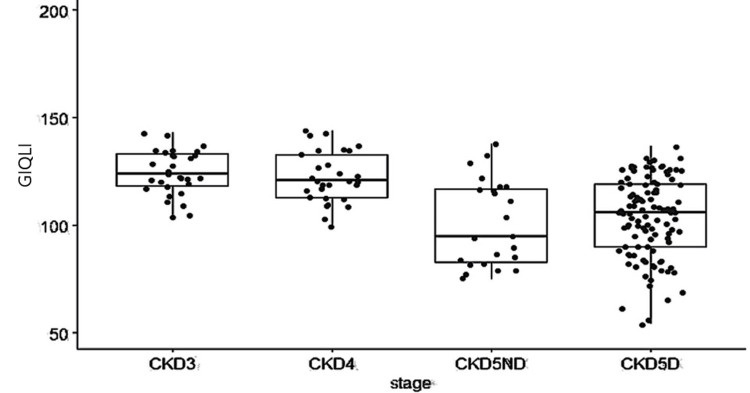
Median GIQLI scores across CKD stages GIQLI scores are compared among patients in CKD stages III, IV, and V not on dialysis (CKD5ND) and CKD stage V on dialysis (CKD5D). Boxes represent the IQR, with medians indicated by horizontal lines. A statistically significant difference in GIQLI scores was observed across CKD stages (p<0.001, Kruskal-Wallis test). GIQLI: Gastrointestinal Quality of Life Index; CKD: chronic kidney disease; IQR: interquartile range

The median GIQLI score was 113 (IQR: 98-124.5). Participants with CKD stages III and IV had higher GIQLI scores (124 (118.5-133.5) and 121 (113-133), respectively) compared to those with stage V CKD. For stage V CKD, participants not receiving hemodialysis had a score of 92 (IQR: 84-104), while those receiving hemodialysis had a score of 108 (IQR: 94.8-119). A statistically significant difference in GIQLI scores was observed across the CKD stages (p<0.001) (Figure 3).

A significant and weak negative correlation was observed between the GIQLI and serum creatinine levels (rho=-0.36; p<0.001). Further, a significant and weak positive correlation was found between the GIQLI and the stage of CKD, as indicated by the eGFR (rho=0.40; p<0.001) (Table 4).

**Table 4:** Spearman's correlation indices n*: sample size; **: statistically significant p-values; BMI: body mass index; GIQLI: Gastrointestinal Quality of Life Index

Independent variable	Dependent variable	n^*^	Spearman's coefficient (rho)	P-value
BMI	GIQLI	202	0	0.10
eGFR (in ml/min/1.73 m^2^)	GIQLI	202	0.40	<0.001^**^
Serum creatinine (in g/l)	GIQLI	202	-0.36	<0.001^**^
Duration on hemodialysis	GIQLI	202	0.11	0.25

## Discussion

In this study, we found a high prevalence and frequency of GIS, affecting 82.2% of participants. The most commonly reported symptoms were anorexia (41.1%), constipation (39.6%), nausea (36.6%), bloating (33.7%), and vomiting (32.2%) (Figure 2). GIQLI was independently associated with GIS and decreased with both hypertension and declining renal function. These findings highlight the substantial burden of GIS among CKD patients, consistent with previous research. For example, Kaze et al. conducted a study in Cameroon and reported a similar prevalence of GIS (87.9%), compared to 82.2% in our study [17]. Conversely, Chong and Tan observed a slightly lower prevalence of 70.7% in patients with end-stage renal disease undergoing hemodialysis [8]. This discrepancy could be due to the inclusion of participants at predialytic stages (III and IV) of CKD, who exhibited fewer GIS than those at end-stage renal disease or on hemodialysis. This may have impacted the overall prevalence. Most importantly, it could reflect an under-dialysis status, as our participants were undergoing two weekly hemodialysis sessions, compared to three weekly sessions in Chong and Tan's study [8]. Although the role of hemodialysis in alleviating GIS, particularly uremic symptoms such as nausea, vomiting, and anorexia, is well-documented [18], it did not improve GIS in patients with end-stage renal disease. This suggests that other factors, such as medications, psychosomatic symptoms, or comorbid conditions, may have contributed to the persistence of these symptoms. Additionally, while hemodialysis may relieve some GIS, it can also induce others. Previous studies have highlighted the prevalence of symptoms like constipation and dyspepsia (including bloating, inappetence, nausea, vomiting, and upper abdominal pain) among hemodialysis patients [6,19]. Our findings underscore the GIS burden in CKD patients and suggest that individuals in sub-Saharan Africa may experience a higher frequency of these symptoms compared to other populations.

The impact of GIS on the QoL of CKD patients remains understudied, with most research focusing on how CKD itself affects QoL or comparing QoL outcomes across different renal replacement therapies [2,10,20,21]. In our study, the presence of GIS was associated with a poor QoL. This is consistent with studies by Chong and Tanand Strid et al., who reported a negative correlation between GIS and QoL [7,8]. Essentially, an increase in GIS leads to a decline in QoL. This could be explained by the fact that GIS worsen as kidney function declines, as demonstrated in our study, ultimately contributing to worse QoL outcomes [20]. However, reverse causation must also be considered, where a poor QoL could contribute to the presence of GIS. Moreover, hypertension was linked to a lower QoL, aligning with existing literature [2,22]. Factors beyond hypertension itself, such as limited access to quality healthcare, scarce resources, and the financial burden, may help explain this relationship, especially in low- and middle-income settings like Cameroon [22]. On the other hand, patients with hypertension may experience GIS, such as constipation, which could further deteriorate their QoL [23]. The complex interplay between hypertension and GIS likely involves the gut microbiome, which is increasingly recognized for its role in both hypertension and gastrointestinal health. Recent studies suggest that the gut not only influences blood pressure regulation but also modulates the risk of hypertension, in addition to affecting other organs like the kidney and brain [24-26]. Consequently, addressing GIS in CKD patients could play a key role in controlling blood pressure and improving overall QoL. Thus, while hypertension contributes to poor QoL, GIS may exacerbate this relationship, leading to further declines in QoL.

Strengths and limitations

Few studies have investigated GIS in predialytic stages of CKD. To the best of our knowledge, this is the first study in sub-Saharan Africa that evaluates GIS in predialytic and dialytic stages and examines their impact on QoL using the GIQLI, a tool specifically designed for this purpose. This research highlights the effect of GIS in this patient population and sets the stage for future studies, emphasizing the need for QoL assessments in routine clinical practice to improve patient outcomes.

However, several limitations should be acknowledged. Despite efforts to reduce bias and confounding variables, such as age and sex, the study may still be susceptible to these factors. The cross-sectional nature of the study prevents the establishment of causal relationships between GIS and QoL. Further, caution is needed when interpreting the results due to the potential for reverse causation. A prospective study would be valuable in addressing these limitations and better understanding causal pathways. Additionally, the study's external validity may be limited, as it is primarily applicable to urban populations seeking medical care. Moreover, the use of a 5% absolute margin of error in the sample size calculation may have resulted in reduced precision, particularly given the relatively low expected prevalence of 14%. Future studies may consider smaller margins to enhance accuracy. Lastly, only a subset of GIS from the R4DQ was assessed. While this was a pragmatic decision, it introduces the possibility that unmeasured symptoms could also contribute to QoL impairment. Future studies should consider using the full questionnaire for a more comprehensive assessment.

## Conclusions

Our findings highlight the substantial burden of GIS in CKD and emphasize the need for a more comprehensive approach to their management. Addressing these symptoms goes beyond symptom relief; it requires a deeper understanding of the underlying pathophysiological mechanisms. Hypertension and declining renal function were found to negatively impact gastrointestinal QoL, illustrating the importance of effective blood pressure control. Additionally, incorporating QoL assessments into routine clinical practice is crucial for improving patient health outcomes.

## Appendices

Appendix 1

Rome IV Diagnostic Questionnaire (R4DQ)

The R4DQ [13] can be accessed at https://theromefoundation.org/wp-content/uploads/Rome-Foundation-DGBI-rev.pdf.

Appendix 2

Gastrointestinal Quality of Life Index (GIQLI) Questionnaire

The GIQLI (freely available for academic and non-funded studies) [14] can be accessed at https://drive.google.com/file/d/1zLJCQDJozr9uJdF2_BL0o29FUwbDTXeg/view?usp=sharing and https://eprovide.mapi-trust.org/instruments/gastrointestinal-quality-of-life-index#contact_and_conditions_of_use.
